# Cruelty in Irish Parents

**Published:** 1893-11-04

**Authors:** 


### CRUELTY IN IRISH PARENTS.

Dr. Edward Berdoe writes: "While agreeing with you that the cruelty of the Phelans is due neither to their 'Irish blood' nor their 'Romish religion,' I must take exception, as an Englishman, to the base suggestion of the first of these as factors in the case. It was stated in several of the morning papers that one of the parents is a Protestant and the other a Catholic; both are of course utterly unworthy to be considered Christians in any sense. You ask, 'What then is it?' I venture to suggest that an imperfect religious education and an entire misconception of Christianity itself is the chief reason why so many 'religious' persons treat children with the grossest barbarity. I have known more cases of cruelty to children amongst 'religious' people than amongst the non-professing classes, and I think this is chiefly attributable to the belief that disobedience, ill-temper, wilfulness, and perversity, so common in young children, is (*sic*) the work of the devil, and only to be expelled by violence. The demon theory of disease which possesses savages finds its analagon with us in the demon theory of haughtiness; and exorcism by scourging and other forms of corporal punishment is considered by the most orthodox as the appropriate treatment. Medical men are never half so severe to children as are clergymen, and I find as a rule that the severity of the clerics increases in direct proportion to their orthodoxy. I may be wrong, but I offer you this reply to your question for what it is worth. I was most religiously brought up, but I was most cruelly scourged for the most trifling offences."

